# The simultaneous occurrence of paraganglioma, Takotsubo syndrome, and Markis type I coronary artery ectasia in the same patient is a rare, high-risk clinical syndrome: a case report

**DOI:** 10.1186/s12872-023-03577-1

**Published:** 2023-11-03

**Authors:** Bofeng Chai, Yiping Su, Na Fu, Yuhong Li, Youlu Shen

**Affiliations:** 1 Graduate School of Qinghai University, No. 251 Ningda Road, Xining, 810016 China; 2 Qinghai University Affiliated Hospital, No. 29 Tongren Road, Chengxi District, Xining, 810001 China

**Keywords:** Paraganglioma, Coronary artery ectasia, Takotsubo syndrome, Case report, Phenoxybenzamine, Diagnostic imaging

## Abstract

**Background:**

Population-wide, paraganglioma (PGL) is uncommon. The incidence of Takotsubo syndrome (TTS) ranges from 0.5% to 0.9% and also is an exceedingly rare manifestation of PGL. Coronary artery ectasia (CAE) is also uncommon, with an incidence ranging from 1.2% to 4.9%. Herein, we present a case of PGL, TTS, and Markis type I CAE that occured in the same patient.

**Case presentation:**

A man in his early 40s was admitted to our hospital with a 16-hour history of abdominal colic. Computed tomography and laboratory examination led to the diagnosis of PGL, coronary angiography led to the diagnosis of Markis type I or Chinese type III CAE, and two echocardiographic examinations led to the diagnosis of TTS. When the patient was treated by phenoxybenzamine instead of surgery for the PGL, his blood pressure and glucose level gradually returned to normal. The CAE was treated by thrombolysis, antiplatelet medications, atorvastatin, and myocardial protection therapies. No symptoms of PGL, CAE, or TTS were seen during a 6-month follow-up, and the patient had an excellent quality of life. We confirmed that phenoxybenzamine was the cause of the TTS because paradoxical systolic motion of the apex, inferior wall, left ventricular anterior wall, and interventricular septum were similarly recovered when the PGL was treated by phenoxybenzamine.

**Conclusions:**

To raise awareness of this illness and prevent misdiagnosis, we have herein presented a case of TTS that was brought on by PGL with Markis type I CAE for clinicians’ reference. In addition, in clinical practice, we should consider the possibility of a concomitant coronary artery disease even if the TTS is caused by a PGL-induced catecholamine surge.

## Background

The sympathetic paravertebral ganglia of the thorax, abdomen, and pelvis produce extra-adrenal chromaffin cells, which are the source of paraganglioma (PGL) [1]. Population-wide, PGL is uncommon, only 0.2% to 0.6% of patients with hypertension have pheochromocytoma [2]. Takotsubo syndrome (TTS) is an exceedingly rare manifestation of pheochromocytoma and paraganglioma (PPGL) [1].

The incidence of TTS, a temporary left ventricular wall failure brought on by physiological or emotional disturbances [3], ranges from 0.5% to 0.9%, approximately 4% to 5% of patients with TTS die during the acute phase [4]. Chest pain, dyspnea, and other symptoms are the main clinical manifestations of TTS, which is similar to acute coronary syndrome (ACS) [5].

Coronary artery ectasia (CAE) increases the risk of acute myocardial infarction because of slow blood flow, intracoronary thrombosis, and aberrant lumen shape and function [6]. CAE is also uncommon, with an incidence ranging from 1.2% to 4.9% [7]. Based on the extent of the lesions, *Markis et al*. classified CAE into four kinds in 1976 [8].

The presence of PGL, TTS, and Markis type I CAE occurring simultaneously in the same patient is a rare clinical occurrence, and we herein present this case in order to decrease the misdiagnosis rate in such cases.

## Case presentation

**Fig. 1:**
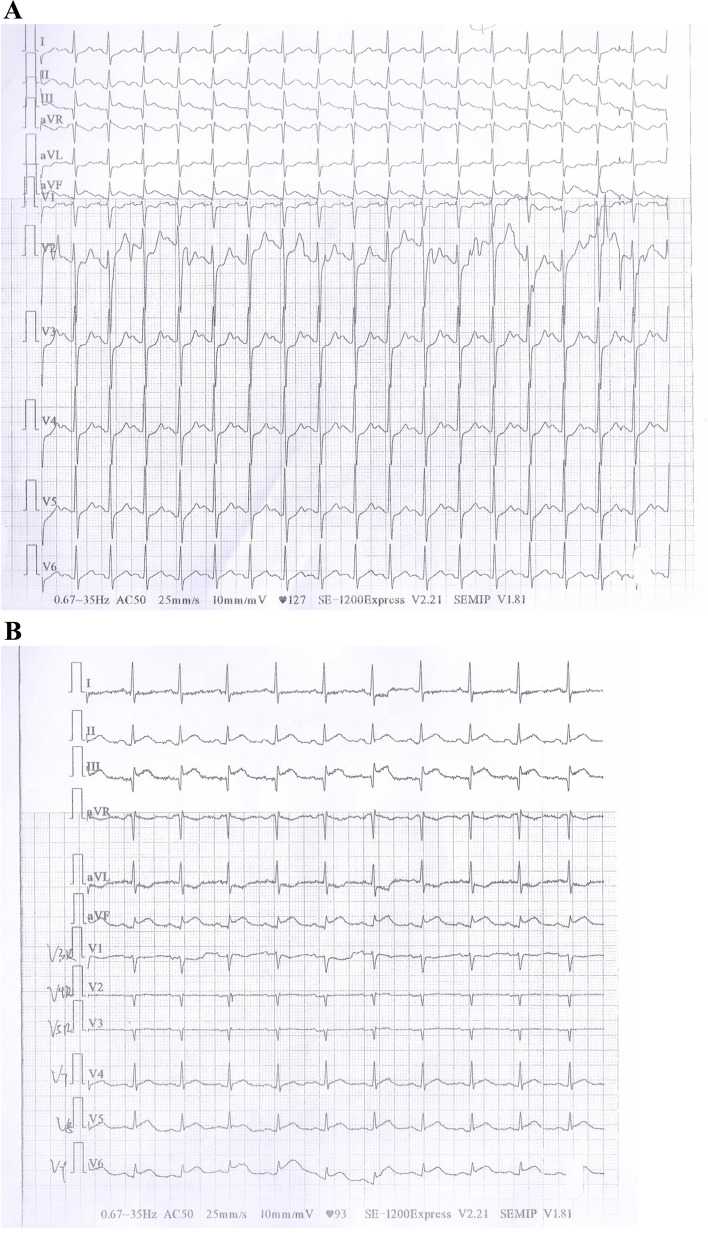
**A**, **B** Emergency electrocardiography at county hospital sinus tachycardia and ST-segment elevation in leads II, III, aVF, V7, V8, and V9

**Fig. 2:**
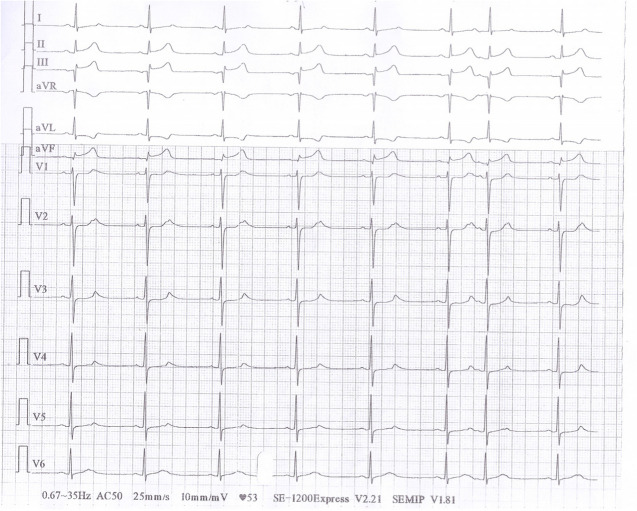
Ectrocardiography after thrombolytic therapy at county hospital. Sinus rhythm, atrial premature beats, and ST-segment elevation in leads II, III, and aVF declined compared to the Fig. 1

A man in his early 40s presented to our hospital’s emergency department with a 16-hour history of continuous stomach colic. Physical examination and questioning at a county hospital revealed that the patient had severe left lower abdominal colic without an apparent cause, along with dizziness, nausea, vomiting, palpitations, and dyspnea. He had no chest pain, cough, sputum production, fever, dysphagia, hematemesis, hematochezia, melena, diarrhea, or abdominal distension. His blood pressure was 210/140 mmHg. Emergency ECG revealed sinus tachycardia and ST-segment elevation in leads II, III, aVF, V7, V8, and V9 (Fig. 1 A, B). The patient was diagnosed with acute myocardial infarction of the inferior and posterior wall. He was treated by 300 mg each of oral aspirin and clopidogrel. With no contraindications to thrombolysis, thrombolytic treatment was also administered, beginning with an intravenous injection of 20 mg of recombinant human prourokinase within 3 minutes and progressing to 30 mg within 30 minutes. After receiving this thrombolytic treatment, the patient experienced a minor improvement in his abdominal colic. Subsequent ECG revealed sinus rhythm, atrial premature beats, and lower ST-segment elevation in leads II, III, and aVF than previously observed (Fig. 2).

**Fig. 3:**
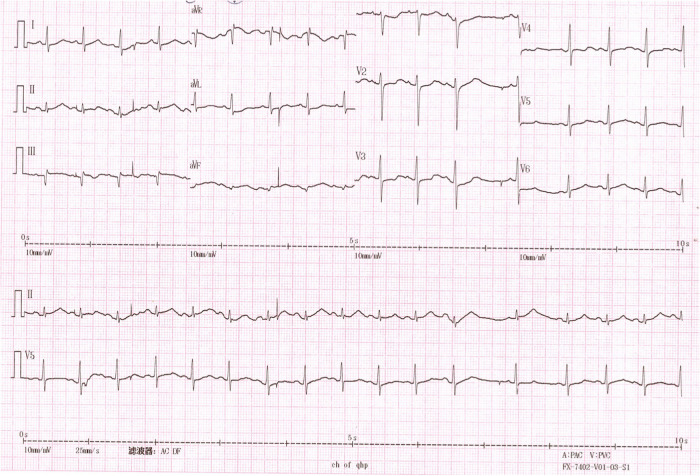
Ectrocardiography at cardiovascular hospital sinus rhythm, with lower ST-segment elevation in leads II, III, aVF, V7, V8, and V9 than in Figs. 1 and 2

Following the patient’s transfer to a cardiovascular hospital, ECG revealed sinus rhythm and lower ST-segment elevation in leads II, III, aVF, V7, V8, and V9 than observed in the first two ECG examinations (Fig. 3). Echocardiography revealed paradoxical systolic motion of the apex, inferior wall, left ventricular anterior wall, and interventricular septum as well as a left ventricular end-diastolic diameter of 57 mm and a left ventricular ejection fraction of 35%. As shown in Table 1, the levels of the myocardial enzymes cardiac troponin-I and creatine kinase-MB were higher than the reference range. Table 2 shows the blood pressure changes that occurred during the patient’s stay in the cardiovascular hospital; his blood pressure was higher than the reference range on four instances. The same diagnosis of acute myocardial infarction was made, and nitroglycerin was administered for treatment.

**Table 1:** Results of myocardial enzymology at Cardiovascular Hospital

**CK** ng/ml	**CK-MB**(0-5ng/ml)	**Myo**(0-70ng/ml)	**cTNI** (0-0.1ng/ml)
/	19.11	<30	24.17

*CK* CreatineKinase, *CK-MB* CreatineKinase-MB, *Myo* Myohemoglobin, *cTNI* Cardiac troponin-I

**Table 2:** Blood pressure changed during cardiovascular hospital

**Time**	19:30	19:40	20:30	21:30	22:30
**BP(mmHg)**	134/94	160/103	151/97	143/87	120/82

*BP* Blood Pressure

**Fig. 4:**
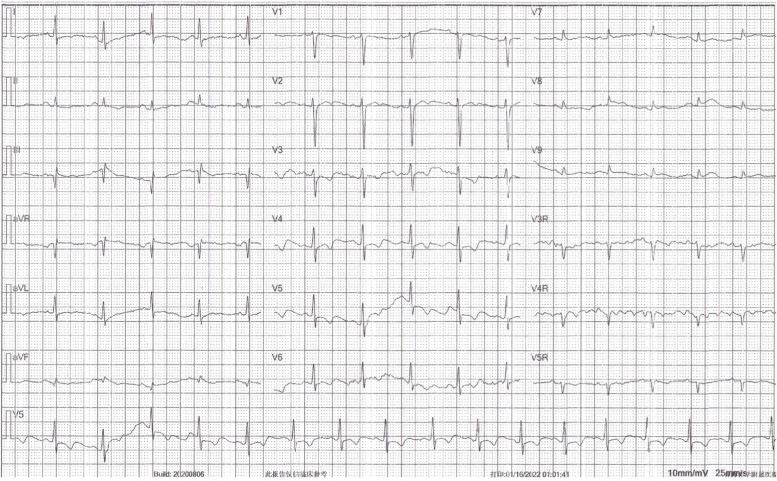
Ectrocardiography at Emergency Department of our hospital elevated ST- segment returning to baseline, Q-wave deepening in lead III, Q-wave forming in lead aVF, and inverted T-waves in leads V3, V4, V5, and V6

**Fig. 5:**
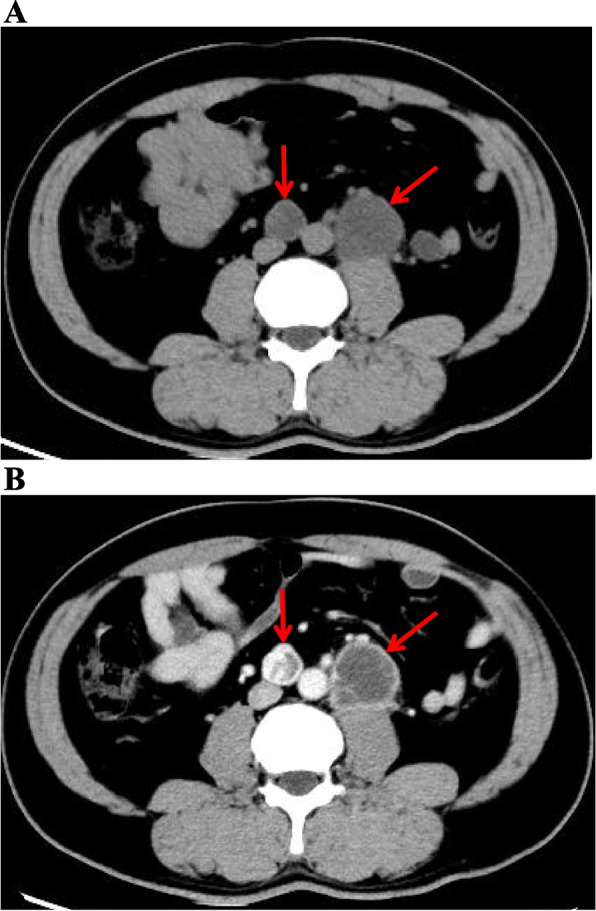
**A** and **B** Abdominal computer tomography plain and enhanced scan. Masses and mixed low-density shadow on both sides of the abdominal aorta, and marked with a red arrow. The larger mass was located on the left side and measured 4.01×3.94cm; it was visible thin walls and partitions; the CT value was approximately 14HU; and the enhanced scan revealed annular enhancement. CT: computer tomography

**Fig. 6:**
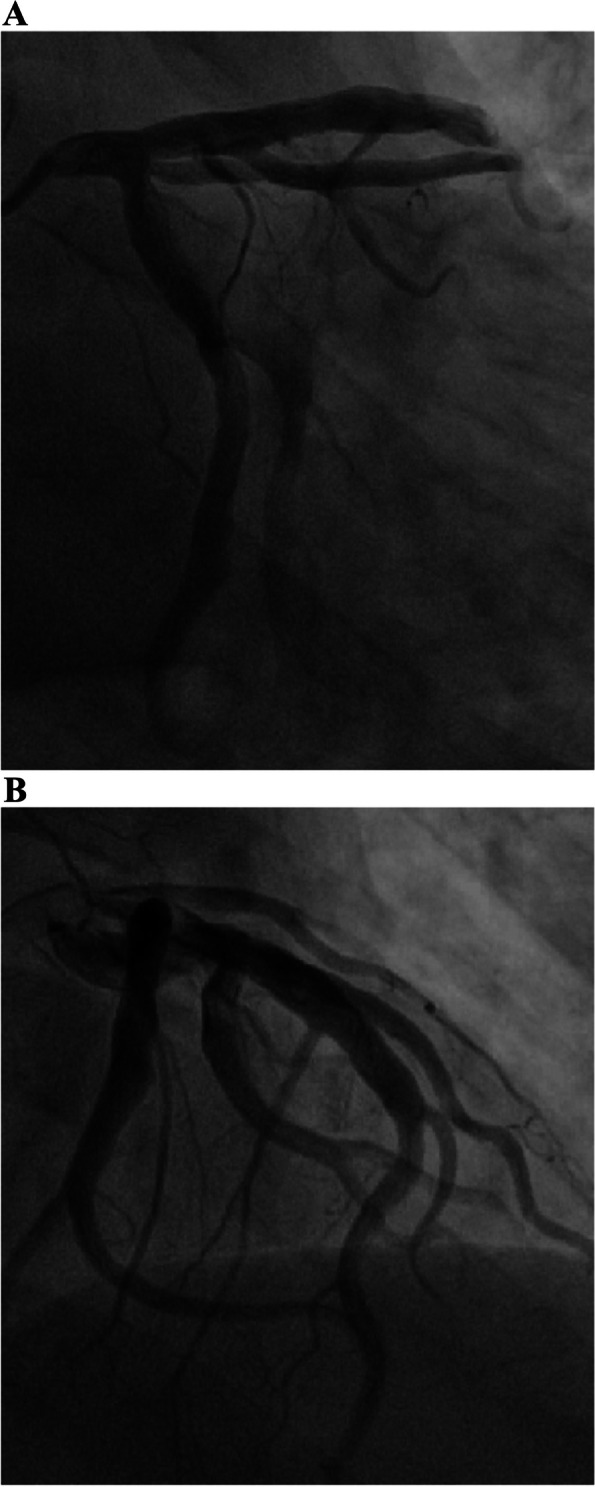
**A** and **B** Coronary angiography on the fourth day of hospitalization. Left main (LM) had no obvious stenosis; Proximal and middle segment of left anterior descending (LAD) artery and left circumflex (LCX) artery had diffuse ectasia with rough endangium, forward blood flow grade TIMI 2, conformed to Markis type I diagnosis standard; The obtuse marginal (OM) artery had irregular stenosis and forward blood flow grade TIMI 2

The patient was then sent to our hospital’s emergency department. Figure 4 shows the results of the ECG examination on admission: elevated ST-segment returning to baseline; Q-wave deepening in lead III; Q-wave forming in lead IVF; and inverted T-waves in leads V3, V4, V5, and V6. The patient’s medical history included 3 years of paroxysmal hypertension (maximum blood pressure of 230/146 mm Hg) and 10 months of undiagnosed hyperglycemia. Assessment of his vital signs revealed a temperature of 36.5°C, heart rate of 94 beats/minute, respiratory rate of 20 breaths/minute, and blood pressure of 100/64 mmHg. Physical examination revealed left lower abdominal pain, cyanosis of the lips, low breath tones in both lungs, and a slight amount of moist rales. Figure 5 shows the emergency plain and enhanced abdominal computed tomography scans, which revealed masses and mixed low-density shadows on both sides of the abdominal aorta. The larger mass was located on the left side and measured 4.01×3.94 cm, had visibly thin walls and partitions, and had a computed tomography value of approximately 14 HU. The enhanced scan revealed annular enhancement of this mass, which was classified as PGL. Emergency coronary angiography, shown in Fig. 6, revealed the following: Left main (LM) had no obvious stenosis; proximal and middle segment of left anterior descending (LAD) artery and left circumflex (LCX) artery had diffuse ectasia with rough endangium, forward blood flow grade TIMI 2, conformed to Markis type I diagnosis standard [8]. The obtuse marginal (OM) artery had irregular stenosis and forward blood flow grade TIMI 2. The patient ultimately received thrombolysis agents, antiplatelet therapy, atorvastatin, and myocardial protection agents. His glycosylated hemoglobin A1c concentration was 10.3%, and an oral glucose tolerance test revealed that his plasma glucose level was 15.0 mmol/L after 2 hours (Table 3). As a result, we were able to determine that he had diabetes mellitus, though it was likely secondary. The results of the laboratory tests for PGL are shown in Table 4. The patient’s free normetanephrine level was significantly elevated in both plasma and 24-hour urine, his normetanephrine + metanephrine level was elevated in plasma, and his 3-methoxytyramine and vanillylmandelic acid levels were elevated in urine. PGL was diagnosed and treated by phenoxybenzamine; surgery was recommended, but the patient declined. Table 5 shows the results of myocardial enzymology performed while the patient was hospitalized. The patient’s health gradually but clearly improved. His systolic blood pressure ranged from 104 to 136 mmHg, and his diastolic blood pressure fluctuated between 66 and 82 mmHg during the 6-month post-discharge follow-up. His fasting plasma glucose level varied from 4.2 to 5.8 mmol/L, and another oral glucose tolerance test showed that his plasma glucose level after 2 hours was <11.0 mmol/L. Another physical examination revealed no cyanosis of the lips, normal breath tones in both lungs, no moist rales, no discomfort in the left lower abdomen, and no other bothersome symptoms, all indicating a good overall clinical condition. Another echocardiography examination after 6 months revealed normal systolic motion of the apex, inferior wall, left ventricular anterior wall, and interventricular septum; a left ventricular end-diastolic diameter of 50 mm; and a left ventricular ejection fraction of 66%. Therefore, according to the European guidelines [9], the patient had no red flags of acute infectious myocarditis, and was retrospectively diagnosed with TTS combined with the echocardiographical findings at the cardiovascular hospital and the after 6 months of follow-up. Finally, we were able to validate the efficacy of phenoxybenzamine for the PGL; thrombolysis, antiplatelet therapy, and lipid modulation for the CAE; and myocardial protection for the TTS. Consent for all treatments was obtained from the patient. He was ultimately diagnosed with TTS produced by PGL combined with Markis type I CAE.

**Table 3:** Results of the OGTT

**Time(h)**	**Fasting**	**1h**	**2h**	**3h**
**GLU ( mmol/L)**	5.2	10.2	15.0	16.5

*OGTT* Oral Glucose Tolerance Test, *GLU* Glucose

**Table 4:** Laboratory examination of paraganglioma

**Samples**	**Plasma** (Reference range)	**Urine** (Reference range)
**Free NMN**	>1000 **↑** (<145pg/ml)	3070.7 **↑**(125-510ug/24h)
**Free MN**	13.1 (<62pg/ml)	85.4 (62-270ug/24h)
**NMN+MN**	>1013.1 **↑** (<207pg/ml)	
**Free E**	/	5.3
**Free NE**	/	733.6
**Free DA**	/	545
**3-MT**	/	320.3 **↑** (≤306ug/24h)
**VMA**	/	12mg **↑ **(0-7.5mg/24h)
**HVA**	/	5.9 (0-10mg/24h)
**Urine volume in 24 hours**	/	2660mL

*NMN* Normetanephrine, *MN* Metanephrine, *E* Epinephrine, *NE* Norepinephrine; *DA*: Dopamine, *3-MT* 3-methoxytyramine, *VMA* Vanillylmandelice acid, *HVA* Homovanillic acid. Above normal range of indices were indicated by an arrow

**Table 5:** Results of myocardial enzymology examination during hospitalization

	**CK** (50-310)U/L	**CK-MB** <24U/L	**Myo** <70ng/ml	**cTNI** <0.023ng/ml	**NT-proBNP** <450ng/L	**D-dimer** (0-1)mg/L
**ED**	/	43	63	1.8	1320	2.16
**January 16**	347	34	31	1.5	2060	1.5
**January 17**	131	23	45	0.55	1100	0.6
**January 18**	65	13	44	0.23	612	/
**January 20**	37	18	21	/	/	0.7

*CK* CreatineKinase, *CK-MB* CreatineKinase-MB, *Myo* Myohemoglobin, *cTNI* Cardiac troponin-I, *NT-proBNP* N-terminal pro-B-type natriuretic peptide, *ED* Emergency department

## Discussion and conclusions

Endocrine hypertension is brought on by PPGL, a neuroendocrine tumor [1]. PPGL produces one or more CAs, such as epinephrine, norepinephrine, and dopamine, which can enhance sympathetic stimulation and have fatal effects on the heart, brain, kidneys, and blood vessels [1]. Because all PPGLs have the ability to spread, the categorization of benign and malignant PPGL was replaced by metastatic and non-metastatic, respectively. A lesion is described as metastatic if it affects non-chromaffin tissues such as bone, liver, lung, lymph nodes, brain, or other tissues [10]. In addition to radical surgery, several medications are used in the treatment of PPGL, including phenoxybenzamine, phentolamine, cyclophosphamide, cisplatin, sunitinib, and octreotide [10]. In the present case, the patient had secondary diabetes mellitus and typical paroxysmal hypertension. Even if two PGLs are present on both sides of the abdominal aorta, we should consider the chance that the PGLs originated in the heart [11]. Our patient declined surgery and was treated by phenoxybenzamine. After 6 months, his blood pressure and glucose levels returned to normal, further supporting our diagnosis.

As early as 1990, the Japanese scholar *Sato* reported the first case of TTS [12], also known as Takotsubo cardiomyopathy or stress cardiomyopathy. The clinical symptoms of TTS are similar to those of ACS. Left ventricular outflow tract blockage, endothelial dysfunction, sympathetic hyperexcitability, CA toxicity, coronary spasm, and estrogen deficiency are among the research hotspots for investigating the pathogenesis of TTS [5, 13–15]. It has been suggested that apical high *β-* adrenoceptors cause apical hypocontractility during epinephrine overflow [16]. In a study by Wittstein et al. [17], patients with TTS had plasma CA concentrations that were several times higher in the acute phase than those of patients with ST-elevation myocardial infarction. We have reason to suspect that phenoxybenzamine was the source of TTS in this case because the paradoxical systolic motion of the apex, inferior wall, left ventricular anterior wall, and interventricular septum were all restored after PGL was treated by this drug. Cardiac magnetic resonance imaging is indeed a first-line diagnostic tool for the assessment of TTS [18], but our patient refused to undergo this examination because of his fear of magnetic resonance imaging.

Coronary ectasia is defined as a coronary artery diameter >1.5 times that of the neighboring normal coronary artery segments. It is classified as diffuse when the ectasia range exceeds 50% of the coronary artery length and when a coronary aneurysm has developed [19]. According to one report [20], the causes of CAE include atherosclerosis (50%), Kawasaki disease, polyarteritis nodosa, autoimmune disease, infection, trauma, and congenital malformation. Autopsy and pathology findings may include transmural inflammation with lymphocytes, neutrophils, macrophages, and eosinophils as well as a distended thrombus within the ectasia [19]. CAE is characterized by slow blood flow, blood stasis, easy spasm, and easy thrombosis leading to microcirculation disturbance [19]. Losartan (an angiotensin II type 1 receptor antagonist), statins, antiplatelet agents, anticoagulation therapy, stenting, and coil embolization are nonsurgical treatments for CAE, whereas percutaneous coronary intervention and coronary artery bypass grafting are surgical treatments for CAE [19]. In 2018, Chinese researchers reclassified CAE into five types [21], which improved guided treatment [22]. In the present case, the Markis type I [8] or Chinese type III [21] classification was utilized, and the patient was treated according to the Chinese guidelines [21]: thrombolysis, antiplatelet therapy, atorvastatin, and myocardial protective treatments. Although thrombolytics was used in this case in the county hospital, we should strictly follow its contraindications and indications. No ACS-associated angina had occurred by 6 months after discharge.

There is considerable evidence that sympathetic stimulation is central to TTS’s pathogenesis [23], and myocardial stunning is its main clinical characteristic. Among the many mechanisms leading to myocardial stunning, microcirculation disturbance plays an important role [23]. Because of this, we should assume that both PGL and CAE were responsible for the patient's TTS rather than just one of them.

The simultaneous occurrence of PGL, TTS, and Markis type I CAE in the same patient is a rare, high-risk clinical syndrome. To raise awareness of this illness and prevent misdiagnosis, we have herein presented this case for clinicians’ reference. In addition, in clinical practice, we should consider the possibility of a concomitant coronary artery disease even if the TTS is caused by a PGL-induced CA surge [24]. Although this is a high-risk clinical syndrome, we should limit the extension of the presentation of the case as it is only a case report after all.

## Data Availability

Not applicable.

## Abbreviations

PGL: Paraganglioma
PPGL: Pheochromocytoma and paraganglioma
CAE: Coronary artery ectasia
TTS: Takotsubo syndrome
STEMI: St-segment elevation myocardial infarction
ECG: Electrocardiogram
ACS: Acute coronary Syndrome
LAD: Left anterior descending
LCX: Left circumflex artery
LVEDD: Left ventricular end-diastolic diameter
RCA: Right coronary artery
LVEF: Left ventricular ejection fraction
CT: Computer tomography
NMN: Normetanephrine
MN: Metanephrine
3-MT: 3-methoxytyramine
VMA: Vanillylmandelice acid
NT-proBNP: N-terminal pro-B-type natriuretic peptide
BNP: Brain natriuretic peptide
CK: CreatineKinase
CK-MB: CreatineKinase-MB
Myo: Myohemoglobin
cTNI: Cardiac troponin-I
OM: Obtuse marginal
OGTT: Oral Glucose Tolerance Test
E: Epinephrine
NE: Norepinephrine
DA: Dopamine
HVA: Homovanillic acid
CA: Catecholamine
CMRI: Cardiac magnetic resonance imaging
CAD: Coronary artery disease
